# Antitumor Activity of Rosmarinic Acid-Loaded Silk Fibroin Nanoparticles on HeLa and MCF-7 Cells

**DOI:** 10.3390/polym13183169

**Published:** 2021-09-18

**Authors:** Marta G. Fuster, Guzmán Carissimi, Mercedes G. Montalbán, Gloria Víllora

**Affiliations:** Chemical Engineering Department, Faculty of Chemistry, Regional Campus of International Excellence “Campus Mare Nostrum”, University of Murcia, 30071 Murcia, Spain; marta.g.f@um.es (M.G.F.); guzmanaugusto.carissimin@um.es (G.C.); gvillora@um.es (G.V.)

**Keywords:** rosmarinic acid, silk fibroin, nanoparticle, antitumor activity, cell viability, cellular uptake, cell cycle, apoptosis

## Abstract

Rosmarinic acid (RA), one of the most important polyphenol-based antioxidants, has drawn increasing attention because of its remarkable bioactive properties, including anti-inflammatory, anticancer and antibacterial activities. The aim of this study was to synthesize and characterize RA-loaded silk fibroin nanoparticles (RA-SFNs) in terms of their physical–chemical features and composition, and to investigate their antitumor activity against human cervical carcinoma and breast cancer cell lines (HeLa and MCF-7). Compared with the free form, RA bioavailability was enhanced when the drug was adsorbed onto the surface of the silk fibroin nanoparticles (SFNs). The resulting particle diameter was 255 nm, with a polydispersity index of 0.187, and the Z-potential was −17 mV. The drug loading content of the RA-SFNs was 9.4 wt.%. Evaluation of the in vitro drug release of RA from RA-SFNs pointed to a rapid release in physiological conditions (50% of the total drug content was released in 0.5 h). Unloaded SFNs exhibited good biocompatibility, with no significant cytotoxicity observed during the first 48 h against HeLa and MCF-7 cancer cells. In contrast, cell death increased in a concentration-dependent manner after treatment with RA-SFNs, reaching an IC_50_ value of 1.568 and 1.377 mg/mL on HeLa and MCF-7, respectively. For both cell lines, the IC_50_ of free RA was higher. The cellular uptake of the nanoparticles studied was increased when RA was loaded on them. The cell cycle and apoptosis studies revealed that RA-SFNs inhibit cell proliferation and induce apoptosis on HeLa and MCF-7 cell lines. It is concluded, therefore, that the RA delivery platform based on SFNs improves the antitumor potential of RA in the case of the above cancers.

## 1. Introduction

During recent years, natural compounds and their derivatives have increasingly been investigated for their curative properties and for their potential in the development of new drugs [1,2,3]. Among these curative properties, the anticancer activity of natural compounds has gained increasing attention as an alternative to the limitations of conventional chemotherapies, i.e., severe side effects and inefficacy due to the multi-drug resistance [1,4].

Rosmarinic acid (RA), an ester of caffeic acid, is a naturally occurring polyphenolic compound that is found especially in medicinal herbs obtained mainly from the *Lamiaceae* family, such as *Rosmarinus officinalis* (rosemary), from which it was formerly isolated [1,2]. RA has been used in Traditional Chinese Medicine since ancient times because of its potent curative activity as a neuroprotective [3], antioxidant [4], anti-inflammatory [5], antimicrobial [6], anti-allergic [7], antimutagenic [8], antivirus [9] and anticarcinogenic [1,2,10,11] agent. In tumor research, RA has been recognized as an efficient candidate to suppress several cancer cells through different ways by intervening in tumor cell proliferation, reducing inflammation, inducing apoptosis and interfering with the signaling pathways involved in the up-regulation of metastasis, such as ERK [2,12,13].

However, the effective use of polyphenols, including RA, for clinical applications is limited due to their poor solubility in water, low bioavailability after administration in vivo and high instability in the intestinal microflora [14,15]. In this regard, the development of appropriate strategies such as novel drug delivery systems to enhance the bioavailability and pharmacokinetics of RA is crucial. The encapsulation of RA in drug nanocarriers is a promising option for increasing the therapeutical performance of RA because the longer circulation times achieved in blood allow prolonged biological activity and a greater probability of accumulating in inflamed tissues, in which vascular permeability is increased [16]. With these purposes in mind, RA has been loaded or encapsulated in solid lipid nanoparticles [14,17,18], natural [15,19,20,21] or synthetic [16,22] polymer nanoparticles and inorganic nanoparticles [23]. Among the different approaches explored to date, nanoparticles based on biopolymers are of particular interest as drug nanocarriers because they are biodegradable, natural and environmentally-friendly [24,25].

Silk fibroin (SF), a natural polymer obtained from *Bombyx mori* cocoons, has a unique combination of mechanical and biological properties, including nontoxicity, excellent biocompatibility and biodegradability [26]. This biomaterial, when formulated as nanoparticles, has great potential as a vehicle for drugs due to its capacity to adsorb, transport and deliver a wide range of bioactive molecules [27]. Other polyphenols such as curcumin [25,28,29], naringenin [24,30], quercetin [31] or resveratrol [32] have been incorporated in silk fibroin nanoparticles (SFNs) and have yielded promising results in terms of enhanced antioxidant, antifungal and antitumor activities compared with the free drugs. In a previous work, our research group developed a novel procedure to produce SFNs from ionic liquid-SF solutions obtained by high-power ultrasounds [27]. This procedure represented a notable improvement over traditional methods of SF dissolution [33,34] mainly as a result of: (i) the negligible vapor pressure and easy recyclability of ionic liquids, which make them a “greener” alternative to organic solvents [35], (ii) the possibility of obtaining high concentrations of a stable SF solution (up to 25 w/w% [27]) and (iii) the general ease with which SF can be dissolved.

Cancer is known as one of the most traumatic and stressful diseases, affecting ten million people every year worldwide [36]. Although significant advances in the fields of therapeutics and diagnostics have been achieved, the development of drug resistance and tumor relapse have increased to a great extent. Recently, nanomedicine has drawing great attention to try to solve the key challenges in cancer treatment. In this way, nanotechnology has been thoroughly studied to develop innovative nanoparticulate systems for the delivery of anticancer drugs [36,37] such as RA, immunotherapy [38] or ferroptosis-based anticancer therapy [39]. During the last years, SFNs have been shown to be promising candidates to overcome drug solubility problems and limited membrane permeability of a great variety of antitumor drugs enhancing their activity, as stated above [40].

In the present work, we describe how rosmarinic acid-loaded SFNs (RA-SFNs) were produced using a simple and environmentally friendly process to dissolve SF and load the drug of interest. RA-SFNs are physically-chemically characterized in terms of their size, zeta potential, morphology, drug loading and drug release kinetics, and their antitumor activity against Hela and MCF-7 cells is evaluated. To the best of our knowledge, this is the first time that a protein, such as SF, has been used as nanocarrier of the drug RA to improve their antitumor activity.

## 2. Materials and Methods

### 2.1. Materials

Silk fibroin of *Bombyx mori* was obtained from the cocoons of silkworms reared in the sericulture facilities of IMIDA (Murcia, Spain) and raised on a diet of fresh *Morus alba* L. leaves. The cocoons were shredded in a mill to a 1 mm particle size and treated to remove the sericin with an aqueous solution of Na_2_CO_3_ (0.05 N), boiling twice for 60 min. After washing with distilled water and air-drying, the resulting material has a bright white cotton-like appearance. The ionic liquid 1-ethyl-3-methylimidazolium acetate ([emim^+^][acetate^−^] (97% purity) was purchased from IoLiTec GmbH (Frankfurt, Germany) and was used without further purification. RA (96% purity) was provided by Sigma-Aldrich (Madrid, Spain). Purified water (18.2 MΩ·cm at 25 °C; from a Millipore Direct-Q1 ultrapure water system, Billerica, MA, USA) was used throughout. All other chemicals and solvents were of analytical grade and were used without further purification.

### 2.2. Formulation of Rosmarinic Acid-Loaded Silk Fibroin Nanoparticles (RA-SFNs)

**Scheme 1:**
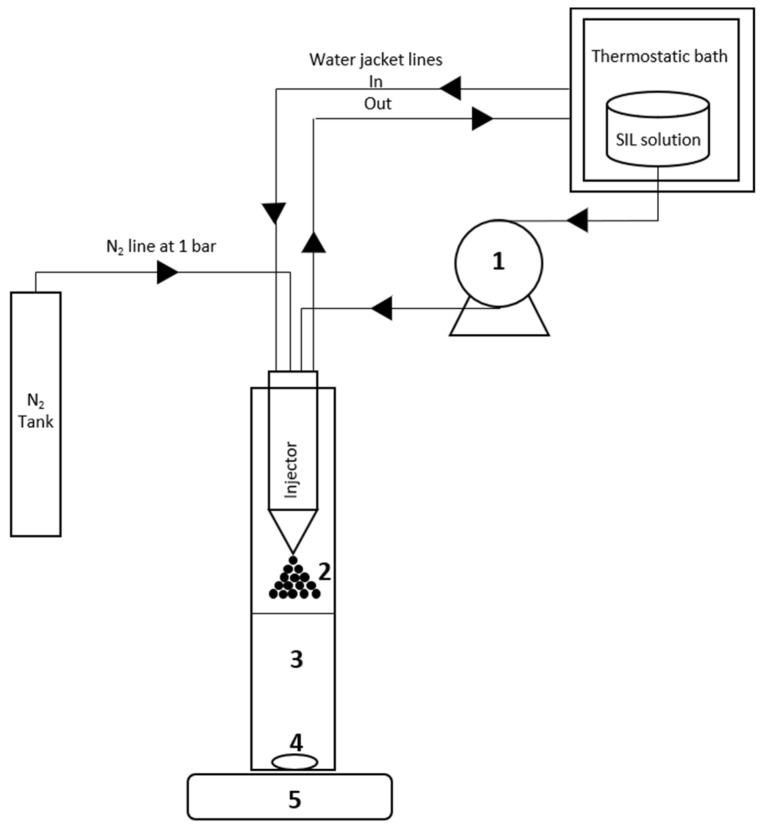
Experimental apparatus used for the SFN synthesis process. (1) Peristaltic pump; (2) SF-[emim^+^][acetate^−^] aerosol generated with pressurized N_2_; (3) Methanol at −20 °C; (4) Stirring bar; (5) Stirring plate [24].

SFNs were prepared following Fuster et al. [24]’s protocol (see Scheme 1). Briefly, an SF-[emim^+^][acetate^−^] solution (10% wt.) was prepared by dissolution with high-power ultrasounds. A program of alternating pulses of 30 s was used for a time of approximately 10 min, with an amplitude of 30% and maximum temperature of 75–80 °C to prevent degradation of the protein. When SF was completely dissolved, 3 mL of ultrapure water was slowly added to this freshly prepared solution to decrease viscosity, obtaining a final SF concentration of 6.66% (wt.) in the solution. The sample was placed in a thermostatic bath at 60 °C and then pumped and precipitated through a thermostatically controlled two-fluid nozzle (from a Mini Spray Dryer B-290, BÜCHI Labortechnik, Flawil, Switzerland, Part No. 044698) into 100 mL of methanol (−20 °C) while stirring vigorously. The feed rate of the SF solution into the spray was maintained at 13.64 mL/min, and the nitrogen pressure was maintained at 1 bar throughout the process. After synthesis, the particles were thoroughly washed with methanol and water to remove the ionic liquid. Finally, the washed particles were resuspended in Milli-Q water and frozen overnight at −20 °C. They were then lyophilized for 72 h, at −55 °C and 0.5 mbar to obtain dry SFNs particles.

To recovery the [emim^+^][acetate^−^] from the methanolic fractions, a BÜCHI RE-111 rotary evaporator, at 80 °C and 80 mbar, was used. The recycled ionic liquid was filtered through 0.22 µm pore diameter, maintained for 24 h in the oven at 100 °C to remove water, and kept in a desiccator under vacuum and phosphorus pentoxide atmosphere until reuse.

For drug loading, 300 mg of SFNs were dispersed in 15 mL of ultrapure water (20 mg/mL) by sonication for 3 min at an amplitude of 30%, with pulses of 5 s ON and 10 s OFF. Next, 6.43 mL of RA (15 mg/mL) dissolved in ethanol were added. The dispersion, with a proportion of 32% *w*/*w* of RA/SFNs, was incubated for 12 h at room temperature protected from light and with orbital shaking. Subsequently, the RA-SFNs were separated from the free RA by centrifugation. Then, the entire supernatant was removed and replaced by ultrapure water. Subsequently, the sample was redispersed by sonication for 3 min at an amplitude of 30% with pulses of 5 s ON and 10 s OFF and analyzed by Attenuated Total Reflectance Fourier Transformed Infrared Spectroscopy (ATR-FTIR, iS5-Nicolet equipped with an iD7 ATR module, Thermo Fischer Scientific, Waltham, MA, USA) to determine RA loading on the particles. Finally, the rest of the dispersion was frozen for subsequent lyophilization.

### 2.3. Physical Characterization of Rosmarinic Acid-Loaded Silk Fibroin Nanoparticles (RA-SFNs)

#### 2.3.1. Size and Size Distribution

The intensity-weighted mean hydrodynamic diameter (Z-average) and Z-potential were measured by dynamic light scattering (DLS) and phase analysis light scattering (PALS) techniques, respectively, using a Malvern Zetasizer Nano ZSP (Malvern Instruments Ltd., Malvern, UK), equipped with a laser of 4 mW power at 633 nm wavelength. It was checked that the samples did not absorb at 633 nm in the above measurement conditions (data not shown). Dispersions of SFNs and RA-SFNs (0.1 mg/mL) were prepared in a 1 mM NaCl solution by 3 min of sonication at an amplitude of 30% with pulses of 5 s ON and 5 s OFF using a Branson Sonifier 450D. The dispersions had a pH of 7.4 and a conductivity of ca. 0.130 mS/cm. The dispersed samples were loaded into a disposable Z-potential cell (DTS1070). The integrated Malvern software calculates the Z-average and size distribution by intensity from the time autocorrelation function of the scattering intensity fluctuation by cumulants analysis and no negative least square, respectively. The size results shown are the average of three measurements, where each measurement consisted of 12 runs of 10 s with no delay between measurements. The velocity at which particles move under an electric field, i.e., their electrophoretic mobility, was measured and later correlated to their Z-potential through Henry’s equation. Smoluchoski approximation (κα = 1.5) was assumed. The electrophoretic mobility and Z-potential results shown are the averages of six measurements taken in a fully automated way by the software with a minimum of 12 runs.

#### 2.3.2. Morphology

The morphological characterization of RA-SFNs was investigated using Field Emission Scanning Electron Microscopy (FESEM) and Transmission Electron Microscopy (TEM). On the one hand, an FEI SciosTM field emission scanning electron microscope (Thermo Scientific, Waltham, MA, USA) operated at 20 kV was used. The sample was placed on a pedestal as powder and then coated with gold. On the other hand, sample preparation for TEM was carried out using 0.06 mg/mL nanoparticle suspensions in purified water and ultrasonification for 3 min with 30% amplitude. A drop of this suspension was placed on a 200-mesh copper grid coated with carbon. Once the drop was dried at room temperature, a drop of uranyl acetate was added and the copper grid was imaged in an FEI TecnaiTM 12 microscope (Thermo Scientific, Waltham, MA, USA) operated at an acceleration voltage of 120 kV.

#### 2.3.3. Drug Loading Determination

The drug loading content (DLC) and encapsulation efficiency (EE) were determined following the method previously developed by Carissimi et al. [30]. Briefly, calibration samples with a known amount of SFNs and RA were prepared from a 15 mg/mL SFNs dispersion and a 5 mg/mL solution of RA. A variable volume of ethanol was added to maintain a ratio of 30% ethanol and 70% water to maintain the RA dissolved and the SFNs properly dispersed.

Before the measurement in ATR-FTIR, the samples were sonicated for 10 s at an amplitude of 10% to ensure their homogeneity. The spectra of the samples were acquired in the form of dry films by placing 1.5 µL of the nanoparticle dispersion on the ATR-crystal and drying it under a gentle stream of air before measuring. For each sample, a total of 32 interferograms were recorded and averaged at a resolution of 4 cm^−1^ with a zero-filling factor of 4, and Fourier-transformed using the Blackman–Harris 3-term apodization function. A background spectrum without a sample with the same number of scans was collected before each measurement.

DLC, defined as in Equation (1), was obtained directly from the calibration line. EE, defined as in Equation (2), was calculated from DLC and the initial mass ratio of RA/SFNs in the loading phase (24%) by a mass balance.

(1) $$\begin{array}{c} \mathrm{DLC}\left(\%\right)=\frac{\mathrm{Mass}\;\mathrm{of}\;\mathrm{RA}\;\mathrm{loaded}\;\mathrm{onto}\;\mathrm{SFNs}}{\mathrm{Mass}\;\mathrm{of}\;\mathrm{RA}-\mathrm{SFNs}}\times 100\; \end{array}$$

(2) $$\begin{array}{c} \mathrm{EE}\left(\%\right)=\frac{\mathrm{Mass}\;\mathrm{of}\;\mathrm{RA}\;\mathrm{loaded}\;\mathrm{onto}\;\mathrm{SFNs}}{\mathrm{Mass}\;\mathrm{RA}\;\mathrm{added}\;\mathrm{to}\;\mathrm{SFNs}}\times 100\; \end{array}$$

#### 2.3.4. Rosmarinic Acid Release from RA-SFNs

In order to study the release behavior of the obtained RA-SFNs, drug release experiments were carried out in phosphate buffered saline (PBS) 1× (pH = 7.4 and pH = 5.4) with 0.5% (*v/v*) of Tween 80. Twenty milligrams of RA-SFNs were dispersed in 1 mL of PBS 1× by ultrasonication and incubated at 37 °C in an Eppendorf tube shaker for 1 day. At predetermined time points (0.5, 1, 1.5, 2, 2.5, 3, 3.5 and 24 h), the samples were centrifuged for 15 min at 13,400 rpm. The concentration of RA in the supernatant was measured by UV-Vis spectrophotometry, for which a calibration curve for RA in PBS had been obtained previously. For this, samples with RA concentrations of 0.2, 0.5, 1.1, 2.1, 4.2, 10.6 and 21.1 µg/mL in PBS were used, and 1mL of fresh PBS was added to the Eppendorf tube, which was placed again in the shaker. The experiments were carried out in triplicate for every sample, and experimental data were fitted using four release kinetic models found in the literature (Zero order, First order, Ritger–Peppas and Higuchi) [24,25,41] to investigate the drug release mechanism.

### 2.4. In Vitro Antitumor Activity

#### 2.4.1. Cell Culture

MCF-7 breast cancer and HeLa cervical cancer cell lines were purchased from the American Type Culture Collection (ATCC, Manassas, VA, USA), validated and stored according to the supplier’s guidelines. Cells were maintained in Dulbecco’s Modified Eagle Medium supplemented with 10% (*v/v*) heat-inactivated fetal bovine serum, 1 mM glutamax, 1 mM pyruvate and 1% penicillin-streptomycin. Cells were cultured in a humidified atmosphere with 5% CO_2_ at 37 °C, and grown in a 75 cm^2^ culture flask. After a few passages, the cells were seeded for the experiments and, when they reached confluence, they were detached using a solution of 0.25% trypsin-EDTA.

#### 2.4.2. Cell Treatment

RA was dissolved in PBS. HeLa and MCF-7 were plated at 15,600 cells/cm^2^. After 24 h, cells were fed with fresh culture medium supplemented with different final concentrations of RA and RA-SFNs. The DLC result of RA-SFNs was taken into account to test loaded nanoparticles and a concentration range of 0.4–3.5 mg/mL was assessed in wells using the same concentration of RA in the RA-SFNs as in the experiments involving free RA. SFN controls were performed at 3.5 mg/mL in order to evaluate whether cell viability decreased due to the effect of SF or RA. PBS controls were performed with the corresponding volume at the highest concentration of RA used to determine whether the solvent was cytotoxic. In each experiment, growth medium without nanoparticles was used as a control.

#### 2.4.3. MTT Assay

The cytotoxic effect of RA and RA-SFNs on HeLa and MCF-7 cells was assessed by MTT assay, for which cells were seeded in 96-well plates at a concentration of 5 × 10^3^ cells/well. Twenty-four hours later, the cells were fed with fresh culture medium supplemented with different final concentrations of RA (0.02, 0.041, 0.081, 0.65 and 1.3 mg/mL) and RA-SFNs (0.4, 0.8, 1.2, 1.6, 2, 2.4, 3 and 3.5 mg/mL) for 48 h. At the end of the treatment period, the media were discarded and 200 µL of MTT (3-(4,5-dimethylthiazol-2-yl)-2,5-diphenyltetrazolium bromide) solution at a final concentration of 1 mg/mL were added and left in darkness for 4 h, after which the MTT was removed and 100 µL of dimethyl sulfoxide (DMSO) were added. Absorbance was measured in a microplate reader (Floustar Omega) spectrophotometer at 560 nm. Each sample was tested in three independent sets.

#### 2.4.4. Nanoparticle Cellular Uptake

To determine the cellular uptake of FITC-labelled SFNs and FITC-labelled RA-SFNs, both cell lines were seeded to a 24-well plate and incubated for 24 h. The culture medium was replaced by fresh medium with FITC-SFNs and FITC-RA-SFNs at an equivalent RA concentration of 0.224 mg/mL. All cells were washed with PBS three times and then digested with trypsin to obtain cell suspensions. Cell-associated fluorescence was quantified by a Becton–Dickinson FACScalibur flow cytometer.

#### 2.4.5. Cell Cycle Arrest Assay

Cell cycle arrest studies were performed on HeLa and MCF-7 cell lines. A total of 2 × 10^5^ cells/well were seeded in 24-well plates and allowed to fix to the plate for 24 h at 37 °C in a 5% CO_2_ and 95% humidity atmosphere. Then, free RA and RA-SFNs used at 0.226 and 2.4 mg/mL, respectively, were incubated for 24 h. Four wells of each cell line were untreated and used and used as control. At this point, fixed cells were removed from the wells with trypsin-EDTA, collected and centrifuged (250× *g*, 10 min). The supernatant was removed, and the cells were washed with phosphate-buffered saline (PBS) and centrifuged (250× *g*, 10 min). After another cycle of centrifugation under the same conditions, the supernatant was removed, and the cells were resuspended in 200 µL of PBS. Subsequently, 1 mL of a PBS (30%)—ethanol (70%) mixed solution was added to the cells. The solution was kept in ice for 30 min. Finally, ethanol was removed by centrifugation. Cells were resuspended in 400 µL of PBS, and 50 µL RNase solution and 50 µL propidium iodide (PI) were added at a final concentration of 0.1 mg/mL and 40 mg/mL, respectively. After stirring, cells were incubated for 30 min in the same conditions as for culture in the dark. The PI fluorescence was measured for each cell in a Becton-Dickinson FACScalibur flow cytometer. In each case 20,000 events were acquired.

#### 2.4.6. Apoptosis

To explore the cell death mechanism provoked by RA and RA-SFNs, apoptosis experiments were performed. A total of 2 × 10^5^ cells were typically seeded in a 24-well plate for 24 h. Then, MCF-7 and HeLa cancer cells with RA and RA-SFNs and their controls were incubated. An apoptosis positive control with 8 μM camptotecin was used. After 24 h, cells were collected and washed twice with PBS as described above (no PBS-ethanol mixture was used in this case except for a necrosis positive control). After removing the PBS, 40 µL of a solution containing Annexin V and PI (Annexin V-Fluos from Roche) and 1 mL of incubation buffer (HEPES 10 nM, NaCl 140 mM, CaCl_2_ 5 mM pH = 7.4) were added to the cell pellet. Cells were resuspended in this solution and left at room temperature in the dark for 15 min. 200 µL of PBS was added immediately before the measurements. This experiment was carried out using a Becton–Dickinson FACScalibur flow cytometer, registering the emission at wavelengths of 620 and 525 nm for PI and Annexin V, respectively. In each case, 10,000 events were acquired.

#### 2.4.7. Cell Morphology

A total of 6 × 10^4^ HeLa or MCF-7 cells were seeded into 12-well plates and, after 24 h, the cells were treated with RA (0.113 and 0.226 mg/mL), RA-SFNs (1.2 and 2.4 mg/mL) and SFNs (2.4 mg/mL). Untreated cells were used as a negative control. Free RA concentrations were used according to the amount of RA in RA-SFNs calculated by their DLC value. After 48 h of treatment, the cells were observed and photographed under a phase-contrast Inverted Microscope Zeiss Axio Observer 7.

### 2.5. Statistical Analysis

Data were presented as mean ± SD (standard deviation), calculated from three independent samples using GraphPad Prism 8.0.1 software (GraphPad Software, San Diego, CA, USA). Since normality (Kolmogorov-Smirnov, *p* > 0.05) and homoscedasticity (Levene, *p* > 0.05) were met, the statistical significance was determined using the parametric test of ANOVA (*p* < 0.05) for the comparison of groups.

## 3. Results and Discussion

### 3.1. Physical-Chemical Characterization of RA-SFNs

#### 3.1.1. Hydrodynamic Size and Z-Potential

**Figure 1:**
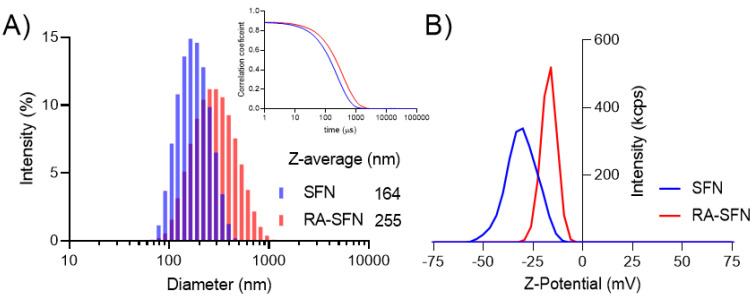
(**A**) Size distribution based on the intensity of SFNs and RA-SFNs with an insert of the correlation coefficient for the two systems and (**B**) Z-potential distribution based on the intensity of SFNs and RA-SFNs.

Figure 1A shows the intensity size distribution of SFNs and RA-SFNs. The raw correlograms from which the size distribution by intensity and Z-average were calculated can be seen in the insert of Figure 1A. As can be seen from the distribution (Figure 1A), both systems present a narrow monomodal distribution with a polydispersity index (PdI) of 0.110 and 0.187 for SFNs and RA-SFNs, respectively. The Z-average and diffusion coefficient values for SFNs were 164 nm and 2.99 µ^2^/s respectively, while the RA-SFNs presented values of 255 nm and 1.93 µ^2^/s, respectively for the same parameters. As regards Z-potential and electrophoretic mobility, SFNs showed a value of −30 mV and −2.408 µmcm/Vs, respectively, while the RA-SFNs presented values of −17 mV and −1.333 µmcm/Vs. Z-potential distribution for the two systems is shown in Figure 1B.

From the results, we can see that the addition of RA to SFNs increases the hydrodynamic diameter and the PdI, and reduces the Z-potential value in absolute terms. These data are consistent with the drug being adsorbed on the surface of the SFNs, thus modifying their chemistry, lowering the Z-potential and increasing their size.

#### 3.1.2. Microscopy

**Figure 2:**
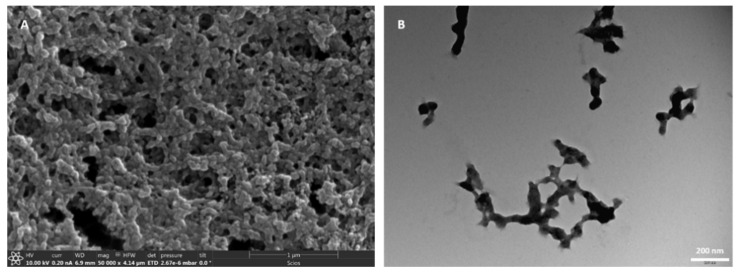
(**A**) Field emission scanning electron microscopy (50,000×) and (**B**) Transmission electron microscopy (46,000×) image of RA-SFNs.

The size and morphology of RA-SFNs were investigated by FESEM and TEM (representative pictures of the system are presented in Figure 2A,B, respectively). It can be seen that the nanoparticles present a granular, quasispheroidal morphology of around 100 nm. When comparing the size observed by microscopy with the Z-average measured by DLS, several things need to be considered. Firstly, in the latter method, size is derived from diffusivity and is given as an intensity weight distribution, while in the former, size is obtained by a number distribution. Secondly, in the case of DLS, particles are dispersed in water whereas they are measured while in FESEM, the particles are dried. Therefore, in DLS a swelling effect due to the protein interaction with water must be borne in mind, as previously described [24,42]. Finally, it is worth noting that DLS size includes the diffusion layer around the particle [43]. Bearing in mind these considerations, the size of RA-SFNs measured by both techniques were in line.

#### 3.1.3. Drug Loading

**Figure 3:**
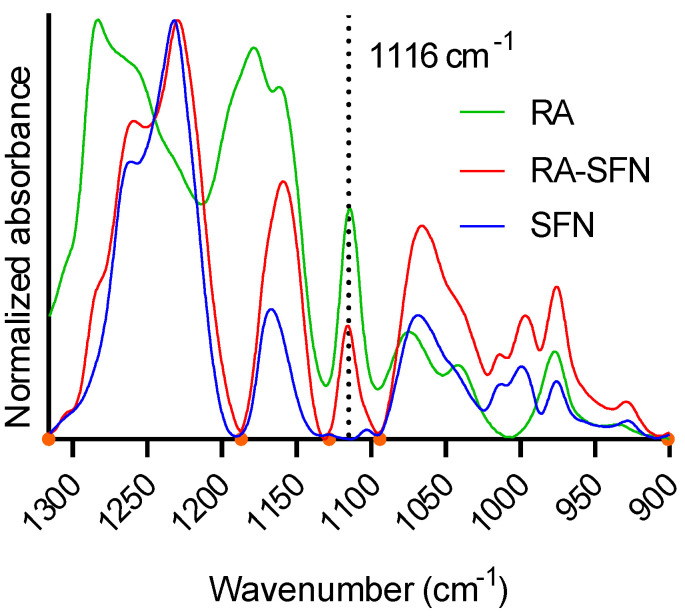
Baseline corrected and normalized infrared absorption spectra of SFNs, RA and RA-SFNs. Spectra are normalized to an absorbance of 1 at the maximum of the amide III band at 1229 cm^−1^. The RA spectrum is normalized to an absorbance of 1 at 1281 cm^−1^. All samples were measured as a dry film obtained from a 30% ethanol dispersion. Orange dots indicate the baseline points.

**Figure 4:**
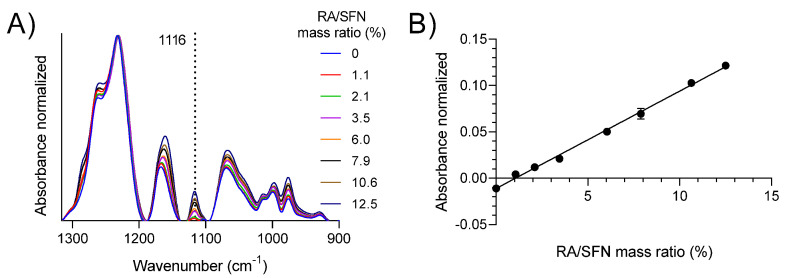
(**A**) Absorbance spectra of calibration samples with constant SFN mass and increasing mass of RA; (**B**) Calibration curve obtained from eight samples measured in triplicate. Some error bars not visible due to the low SD.

The first step for drug quantification, according to the method developed by Carissimi et al. [30], is to identify a signal from the drug in question which shows low-to-no interference with the polymeric matrix of the nanoparticles. From the baseline corrected and normalized ATR-FTIR spectra of RA, SFNs and RA-SFNs (Figure 3), it can be observed that SFNs (blue line) present no signal in the 1137 to 1095 cm^−1^ region, while RA (green line) presents a sharp peak at 1116 cm^−1^. This signal can be also identified in the RA-SFNs (red line) spectrum. It is important to note that no shift is observed. The peak from the amide III band of the SF protein is then used for normalization purposes. A calibration curve was constructed from calibration samples with a constant SFN mass and increasing RA mass. The calibration spectra and calibration curves obtained are presented in Figure 4. From the figure, it can be observed that there is a good linear correlation between the normalized absorbance and the concentration of RA in the sample, with an R^2^ of 0.9968 (as shown in Table 1 along with the curve fitting parameters).

**Figure 5:**
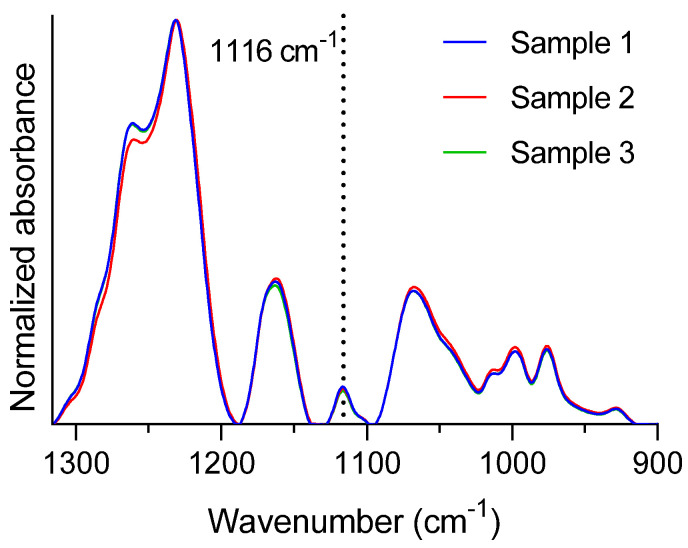
Baseline-corrected, and normalized ATR-FTIR spectra of three RA-SFN samples.

After the loading phase, three independent samples were measured in ATR-FTIR to determine their DLC. Sample spectra are shown in Figure 5. The baseline was corrected, and the spectra were normalized to an absorbance of 1 at 1228 cm^−1^, while the absorbance at 1116 cm^−1^ was interpolated in the calibration curve (Figure 4B). The average DLC obtained was 9.4 ± 0.5% of RA, yielding an EE of 39 %.

#### 3.1.4. Drug Release Kinetics

**Figure 6:**
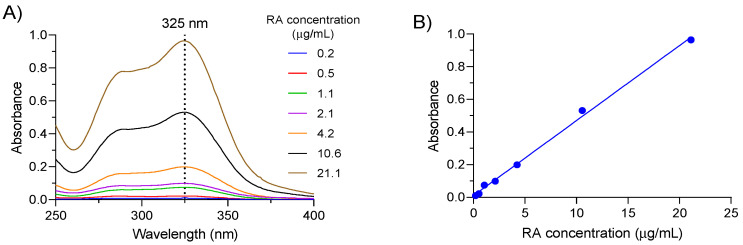
(**A**) UV-vis spectra of calibration samples in PBS. (**B**) Calibration line for RA in PBS. Error bars are not visible due to the low SD.

**Figure 7:**
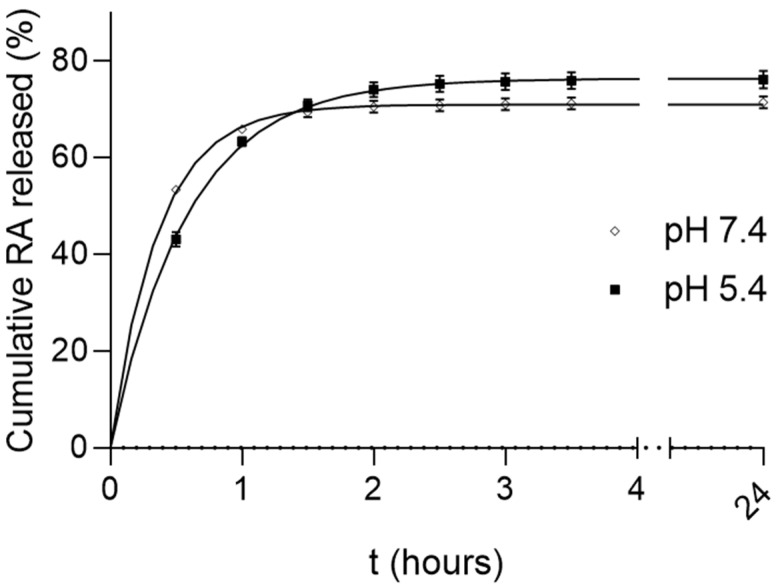
Cumulative release of RA as a function of incubation time in PBS 1× (pH = 7.4 and pH = 5.4) containing 0.5% of Tween 80 at 37 °C. The symbols represent the average experimental values. Continuous lines represent the best fit with a First order equation.

The RA release kinetics of the system was assessed in an in vitro release experiment. PBS with 0.5% *v/v* Tween 80 (PBS_t80_) at pH 7.4 or 5.4 was used as release medium. pH 5.4 was used to simulate the acidic pH of the tumor microenvironment. First, a UV-Vis calibration curve for RA in PBS_t80_ was constructed (see Figure 6), then 20 mg of RA-SFNs (9.4% DLC) were added to Eppendorf tubes with 1 mL of the corresponding release medium and incubated under mild agitation in an oven at 37 °C. At different time points, the particles were centrifuged, the supernatant was extracted for later analysis and the pellet was redispersed by sonication in a fresh release medium and incubated until the next extraction time. For each time point, the released mass of RA was calculated as the value accumulated until this time. As can be seen from Figure 7, the RA release profiles are fast in both cases, i.e., pH 7.4 and pH 5.4. However, the cumulative RA released was slightly lower in acidic conditions than in physiological conditions for the first hour and for the rest of the assay, the RA released was higher in acidic conditions. In summary, we checked that over 40–50% of the drug was released from the particles in 0.5 h, reaching a plateau at around 70–75% at 2 h. Rapid release profiles were also found in previous studies on the encapsulation of RA in polymeric nanoparticles. For instance, da Silva et al. [15] observed that 50 to 60% of RA was released from chitosan nanoparticles at physiological pH in the first 15–20 min.

To ascertain the release kinetics of the system, the experimental data from 0.5 to 2.5 h were fitted to three different drug release models (Zero order, Higuchi and Ritger-Peppas). In the case of First order drug release model, all the experimental data (from 0.5 to 24 h) were used for the fittings due to the excellent fitting goodness. Table 2 shows the fitting equations obtained for each assay and their correlation coefficients. As can be seen in Table 2, the first order equation provided the best fit, and the zero order equation the worst. A similar conclusion was reached for the release kinetics of naringenin from naringenin-loaded SFNs [24]. It is known that some aspects such as the amount of loaded drug and the drug-polymer interaction interfere with the drug release mechanism. Accordingly, a lower release rate when the RA content is decreased seems to indicate that the diffusion through the polymeric phase is the main mechanism of drug release [44].

### 3.2. In Vitro Cytotoxicity

**Figure 8:**
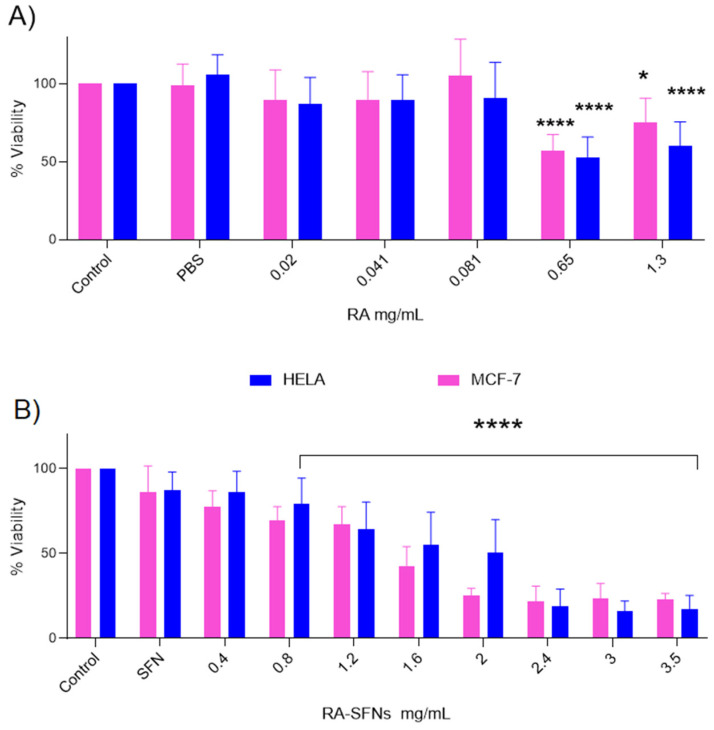
Cytotoxic effect of free RA (**A**), and RA-SFNs (**B**) on HeLa and MCF-7 cell lines. Data are expressed as percentage of cell viability ± SD vs. concentration. * indicates *p* < 0.05, ** indicates *p* < 0.01, *** indicates *p* < 0.001 and **** indicates *p* < 0.0001, compared to control. SFNs without drug were tested at the highest concentration of RA-SFNs (3.5 mg/mL).

The cytotoxic effect of free RA and RA-SFNs on the viability of HeLa and MCF-7 cells was evaluated. The cell lines in question were selected according to their origin and features: HeLa cells are derived from cervical carcinoma and MCF-7 are breast carcinoma cells. Both cell lines are of human origin and have been widely used in cytotoxicity studies. The cells were exposed to different concentrations of RA (0.02–1.3 mg/mL) and RA-SFNs (0.4–3.5 mg/mL) for 48 h, and cell viability was measured by MTT assay. As stated above, the concentration range of free RA was in accordance with the DLC of the RA-SFNs in order to establish a suitable comparison. Figure 8 shows the cell viability as a function of free RA (Figure 8A) or RA-SFN (Figure 8B) concentration. The results show that the cytotoxicity of free RA was much lower against both cell lines than that of RA-SFNs in the same drug concentration range. Indeed, cell viability only decreased to any great extent when a high concentration of free RA was used, (0.65 and 1.3 mg/mL, drug concentrations much higher than that found for the maximum RA-SFN concentration of 3.5 mg/mL, which contained approximately 0.33 mg/mL of RA). However, in the case of RA-SFNs, cell death increased in a concentration-dependent manner: the higher the RA-SFN concentration, the lower the cell viability. The cytotoxicity of unloaded SFNs at the highest concentration of RA-SFNs (3.5 mg/mL) showed no significant differences from the control values.

The results are verified by the IC_50_ values shown in Table 3, which collects the IC_50_ of free RA and RA-SFNs, and also the IC_50_ of RA contained in RA-SFNs, which was calculated from their DLC value, i.e., the amount of RA for each mg/mL of RA-SFNs. The IC_50_ of RA for both cell lines was much lower for RA-SFNs than for free RA, demonstrating the efficacy of RA-SFNs for increasing the bioavailability and, therefore, the efficacy of the antitumor drug.

The antitumor effect of RA on cervical cancer HeLa cells has not been totally elucidated. The previous studies support that RA inhibits the cell cycle by inducing apoptosis [4]. In the case of the breast cancer MCF-7 cell line, previous studies reported that RA inhibited DNA methyltransferase activity [45]. In tumors, aberrant DNA methylation is a mechanism that inactivates tumor suppressor genes, which are as common as mutations. RA may reactivate genes silenced by aberrant methylation through the inhibition of DNA methyltransferase which can be of potential therapeutic value against breast cancer.

### 3.3. Cell Morphology

**Figure 9:**
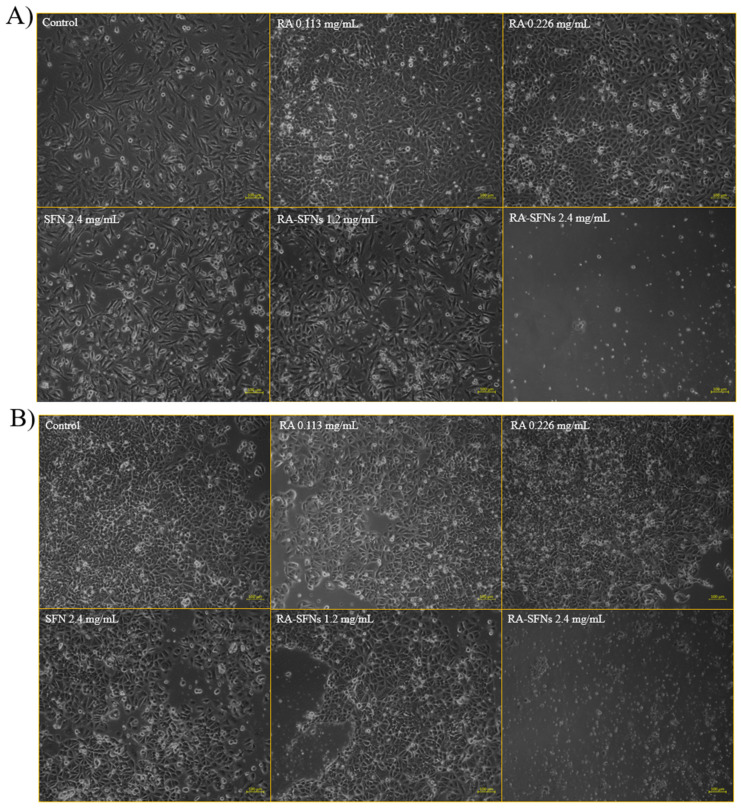
Morphology of (**A**) HeLa and (**B**) MCF-7 cell lines treated with free RA, SFNs and RA-SFNs, and a control (20×).

In order to corroborate the results obtained with the MTT assay, the morphology of HeLa (Figure 9A) and MCF-7 (Figure 9B) cells treated with RA 0.113 mg/mL, RA 0.226 mg/mL, SFNs 2.4 mg/mL, RA-SFNs 1.2 mg/mL and RA-SFNs 2.4 mg/mL was observed by confocal microscopy.

As can be seen in Figure 9, RA-SFNs at a concentration of 2.4 mg/mL led to almost total cell death in both cell lines. However, SFNs at the same concentration only had a slight effect on cell viability, closely reflecting the results of the MTT assay.

### 3.4. In Vitro Cellular Uptake by Flow Cytometry

**Figure 10:**
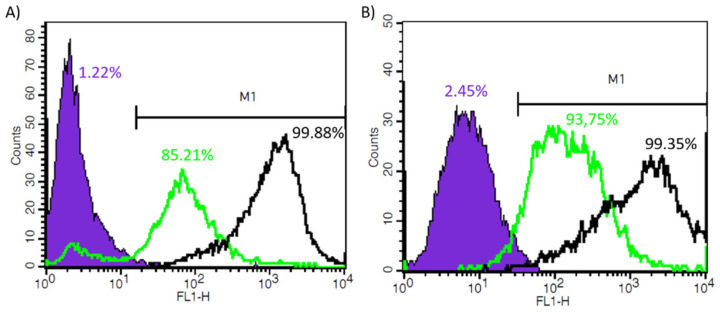
Detection and quantification by flow cytometry of HeLa (**A**) and MCF-7 (**B**) cellular uptake of FITC-labelled SFNs (green) and FITC-labelled RA-SFNs at an equivalent RA concentration of 0.226 mg/mL (black) after 24 h of cell exposure. In both cases, cells without any treatment were used as negative control (purple).

To test the potential of RA-SFNs as possible carriers of RA, we quantified the cellular uptake by determining the median intensity of the fluorescence (FITC) signal in the populations of HeLa and MCF-7 studied using flow cytometry. After culture exposed to 2.4 mg/mL of both FITC-labelled SFNs and FITC-labelled RA-SFNs, median cell fluorescence intensity values increased relative to controls, suggesting that both types of FITC-labelled nanoparticles have been successfully internalized by cells as can be observed in Figure 10. The results showed that the florescence intensity was smaller when cells were exposed to nanoparticles without drug than to FITC-labelled RA-SFNs moving the maximum from 85.21% to 99.88%, respectively, in HeLa cells and from 93.75% to 99.35%, respectively, in MCF-7 cells. These findings confirmed the internalization of both FITC-labelled nanoparticles into HeLa and MCF-7 cell lines, not showing significant differences on the uptake of RA-SFNs in the two cell lines studied. This fact suggests that the cellular uptake and cytotoxicity of the carriers were increased after being grafted with RA.

### 3.5. Influence of Free RA and RA-SFNs Treatment on the Cell Cycle

**Figure 11:**
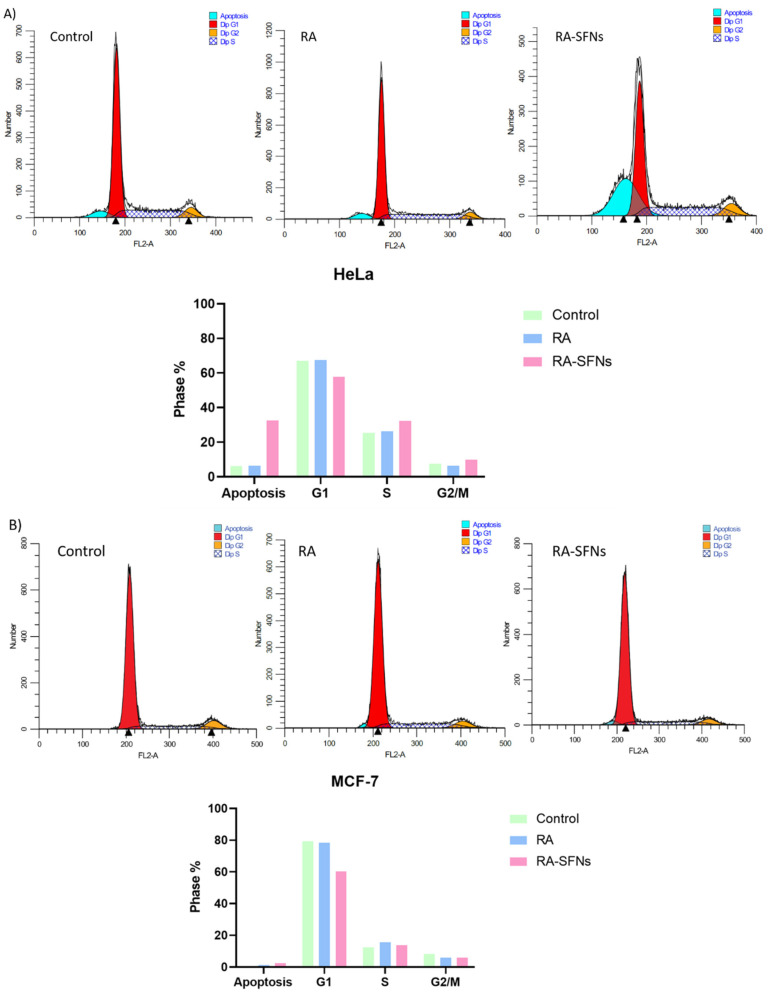
Cell cycle of HeLa (**A**) and MCF-7 (**B**) cells exposed to 0.226 mg/mL of RA and 2.4 mg/mL of RA-SFN for 24 h. Cells not treated were used as control.

To study the inhibition mechanism of cell growth through cell cycle arrest, we examined the effect of 0.226 mg/mL RA and 2.4 mg/mL RA-SFNs (equivalent RA concentration of 0.226 mg/mL) on HeLa and MCF-7 cells for 24 h by flow cytometric analysis. According to Figure 11A, the number of G1 phase in HeLa cells did not change with respect to the control (untreated cells) when cells were treated with free RA, from 67.07% to 67.45%. However, its percentage significantly decreased when cells were exposed to RA-SFNs to 57.70%. The G2/M cell number slightly decreased from 7.59% to 6.32% with free RA but increased to 9.96% with RA-SFNs. The number of cells in the S phase slightly increased from 25.34% to 26.22% with free RA but significantly increased to 32.34% with RA-SFNs. Moreover, we examined the apoptosis phase where the number of cells increased when were exposed to free RA and RA-SFNs from 6.17%(control) to 6.31% and 35.53%, respectively. In summary, a different response to RA and RA-SFNs on HeLa cells has been observed. The effect of the drug RA on HeLa cell cycle progression was much more evident when the drug is incorporated into SFNs suggesting that RA-SFNs cause HeLa cells become arrested in the S phase and then significantly undergo apoptosis rather than proceed to G2/M phase. With the treatment of free RA, the HeLa cell cycle progression was similar to the control.

In MCF-7 cells (Figure 11B), the number of cells in G1 phase was almost constant respect to the control for free RA from 79.32% to 78.29% but again significantly decreased for RA-SFNs to 60.28%. In the case of G2/M phase, the cell number decreased for free RA and for RA-SFNs respect to the control to a same value (from 8.26% to 6.01% and 5.92%, respectively). The S phase cell number increased from 12.41% to 15.70% when exposed to free RA and to 13.80% when RA-SFNs were used. Finally, apoptosis phase was also evaluated. It slightly increased from 0.19% (control) to 1.41% (free RA) and to 2.47% (RA-SFNs). In light of the results of the cell cycle, it seems that RA-SFNs inhibit the cell growth of MCF-7 cells due to the decrease of the percentage of cells in G1 phase.

Several in vitro studies with RA found in the literature have shown the ability of RA to inhibit cell proliferation and induce cell cycle arrest in various cancer cells [10,11]. The apoptosis phase after treatment with RA-SFNs was highly evaluated in the following experiment for both cell lines.

### 3.6. Apoptosis

**Figure 12:**
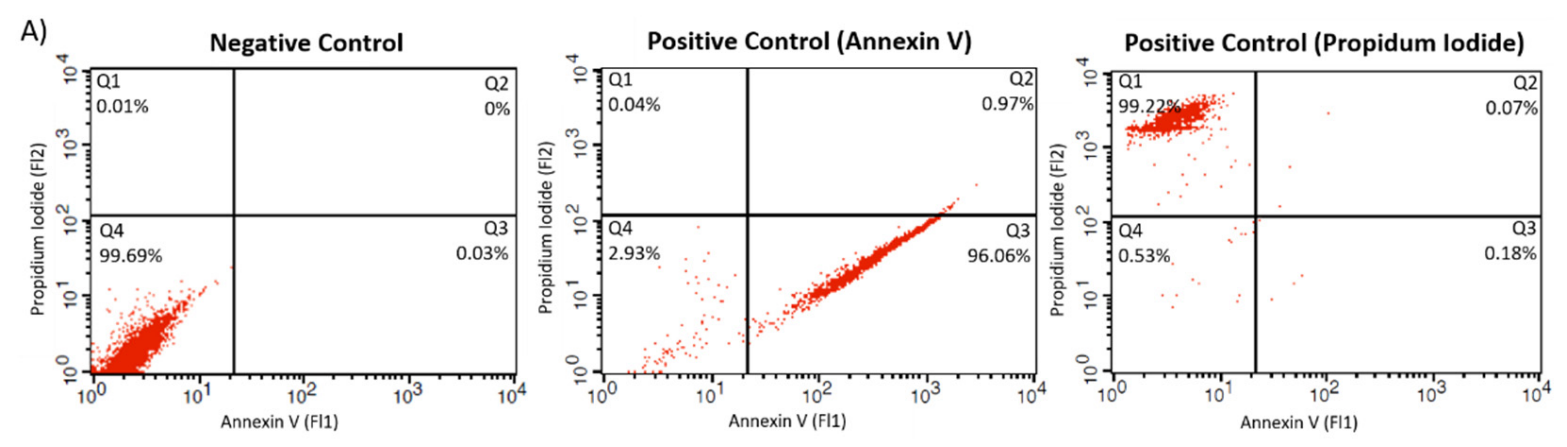

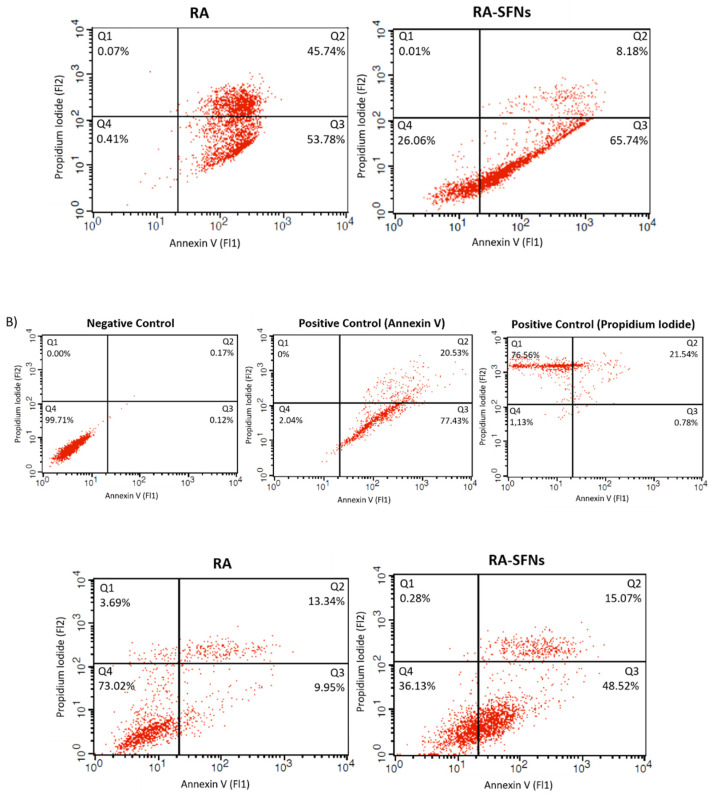
Flow cytometry analysis of apoptosis rate in HeLa (**A**) and MCF-7 (**B**) cells after 24 h of treatment with free RA and RA-SFNs. Cells not treated were used as negative control, cells treated with camptotecin were used as apoptosis positive control (annexin V) and cells not treated, fixed and stained were used as necrosis positive control (propidium iodide).

To analyze the nature of cell death due to the effect of the RA and RA-SFNs on HeLa and MCF-7 cells, Annexin-V-Fluos Apoptosis Detection Kit was used to determine the rate of apoptosis provoked by 24 h of treatment. The Annexin-V-Fluos stained graph consists of four quadrants (Q) where Q1 represents necrotic cells, Q2 and Q3 show late and early apoptotic cells, respectively, and Q4 are the live cells. As negative control live cells which had not been treated were used, the positive apoptosis control, as mentioned in Section 2.4.6, was cells treated with camptotecin, a chemical compound which induces cellular apoptosis and the positive control for necrosis was fixed cells stained with propidium iodide. The positive controls are normally used as comparison and, if similar results are found in the treated cells, it can be assumed that the cells have suffered apoptosis or necrosis. As shown in Figure 12, the apoptotic effect (Q2 + Q3) was quickly observed in HeLa cells compared with MCF-7 cells after treatment with free RA and RA-SFNs. When treated with free RA, 99.52% of the HeLa cells analyzed were in an apoptotic phase, whereas 23.29% of MCF-7 cells exhibited apoptotic effects. On the contrary, the percentage of cells which are dead by necrosis is negligible on both cell lines: 0.07% in HeLa cells and 3.69% in MCF-7 cells. When treated with RA-SFNs, 73.92% of the HeLa cells were in apoptotic phase and 63.59% in MCF-7 cell line. The results about necrosis effects in that case were also insignificant: 0.01% in HeLa cells and 0.28% in MCF-7 cells. In light of the results of the apoptosis analysis, we can conclude that RA-SFNs induce apoptosis on both cell lines leading to a more significant antiproliferative effect on HeLa cells because the increase of the cells on apoptotic phase is higher than in MCF-7 cells. These results are in good agreement with the in vitro cytotoxicity assays which revealed that RA-SFNs in HeLa cells had doble cytotoxic effects than in MCF-7 cells confirming that HeLa cells are more sensitive to RA than MCF-7 cells. These results indicate that RA-SFNs can be regarded as a potential drug targeting cervical and breast cancer. Previous studies have also found that RA induced apoptosis in several cancer cell lines [10,46].

## 4. Conclusions

Nanoparticles are considered advantageous for drug delivery systems. The current study describes the synthesis and characterization of biodegradable RA-SFNs with the aim of improving the bioavailability and, hence, the antitumor activity of the polyphenol RA. The hydrodynamic diameter found for the RA-SFNs was 255 nm, with a narrow size distribution and Z-potential of −17 mV (the values of both parameters suitable for the purpose of the RA-SFNs). In addition, a DLC value of 9.4% and a rapid release efficiency were obtained. An enhanced degree of growth inhibition of RA-SFNs (compared with free RA) against HeLa and MCF-7 was observed in the MTT assay. The relative non-toxicity of the unloaded SFNs indicated good biocompatibility of the nanocarrier. The cellular uptake studies confirmed the total internalization of the RA-SFNs into HeLa and MCF-7 cells after 24 h of treatment, suggesting that the cellular uptake of the nanoparticles was increased after the loading with the drug RA. RA-SFNs induce apoptosis on both cell lines showing a higher antiproliferative effect on HeLa cells, which is in good agreement with the in vitro cytotoxicity assays, thus confirming that HeLa cells are more sensitive to RA than MCF-7 cells. These results indicate that the nanocarrier developed can be used for drug delivery applications, by enhancing the solubility of RA and controlling its release.

## Figures and Tables

**Table 1:** Calibration curve parameters.

	Best-Fit Values	95% Confidence Intervals
Slope	0.0105	0.0099 to 0.0111
Y-intercept	−0.0110	−0.0150 to −0.0069
R^2^	0.9968	

**Table 2:** Fitting parameters for the release models of RA-SFNs.

Model		pH 7.4	pH 5.4
Zero Order	y =	7.93t + 54.11	15.01t + 42.81
	R^2^ =	0.7297	0.8005
First Order	y =	70.99(1−exp(−2.74t))	76.32(1−exp(−1.72t))
	R^2^ =	0.9967	0.9983
Higuchi	y =	19.35t^0.5^ + 43.07	36.30t^0.5^ + 22.41
	R^2^ =	0.8107	0.8743
Ritger–Peppas	y =	62.91t^0.17^	59.25t^0.31^
	R^2^ =	0.8654	0.9002

**Table 3:** IC_50_ values for RA, RA-SFNs and RA contained in RA-SFNs (calculated from DLC value) against HeLa and MCF-7 cell lines as determined by MTT assay. Values were calculated by GraphPad Prism V8.

Cell Line	IC_50_ RA (mg/mL)	IC_50_ RA-SFNs (mg/mL)	IC50 RA in RA-SFNs (mg/mL)
HeLa	1.722	1.568	0.119
MCF-7	3.905	1.377	0.105

## Data Availability

Not applicable.
